# Iodine Adsorption in Nanoporous Carbon to Fabricate Assimilated Battery Electrodes for Durable Hybrid Supercapacitors

**DOI:** 10.3390/ma17143407

**Published:** 2024-07-10

**Authors:** Lucyana Dwi Larasati, Zhazira Supiyeva, Md Tauhidul Islam, Qamar Abbas

**Affiliations:** 1 Department of Metallurgy and Materials Engineering, Faculty of Engineering, Sivas Cumhuriyet University, Sivas 58140, Türkiye; lucyana.d.l@ugm.ac.id; 2 Institute for Chemistry and Technology of Materials, Graz University of Technology, Stremayrgasse 9, 8010 Graz, Austria; zhazira.supiyeva@kaznu.kz (Z.S.); m.islam@tugraz.at (M.T.I.); 3 Faculty of Chemistry and Chemical Technology, Al Farabi Kazakh National University, Al-Farabi Avenue 71, Almaty 050040, Kazakhstan; 4 Institute of Combustion Problems, 172 Bogenbay Batyr Str., Almaty 050012, Kazakhstan; 5 Faculty of Chemical Technology, Institute of Chemistry and Technical Electrochemistry, Poznan University of Technology (PUT), 60965 Poznan, Poland

**Keywords:** hybrid supercapacitor, iodine, aqueous electrolyte, nanoporous carbon, battery electrode, supercapacitor, LiTFSI

## Abstract

A hybrid supercapacitor is designed by coupling a battery electrode with a capacitive electrode in a single device/cell to enhance energy density. In iodine-based hybrid supercapacitors, the nanoporous carbon serves as the electrode material; however, the cathode or positive electrode is charged with iodine via electrodeposition from a redox aqueous electrolyte, while a negative electrode stores charges at the electric double-layer. In this work, iodine is loaded via physical adsorption into the porosity of a carbon electrode, keeping the aqueous electrolyte free from iodide redox moieties. By this way, the risk of polyiodide (I_3_^−^ and I_5_^−^) generation at the positive electrode leading to a shuttling-related performance loss of the hybrid supercapacitor is prevented. Chemical interactions of iodine with the carbon surface and within the pores have been investigated with Raman spectroscopy, thermogravimetry and electron microscopy. Electrochemical methods have been used to test individual electrodes and hybrid supercapacitors in aqueous NaNO_3_ and aqueous LiTFSI at 5 mol/L concentration for performance parameters such as energy efficiency, capacitance, self-discharge and cyclability. The hybrid supercapacitor in aqueous LiTFSI exhibits stable capacitance and energy efficiency during long-term aging tests at 1.5 V. Carbon nanoarchitecturing with iodine as shown in the present work offers an economical approach to enhance the performance of hybrid supercapacitors.

## 1. Introduction

The past few decades have seen a tremendous surge in endeavors to enhance the performance of supercapacitors as a promising form of electrochemical energy storage that bridges the gap between batteries and conventional dielectric capacitors. Supercapacitors are known to quickly harvest and release energy, potentially maintaining an ultra-long lifespan and delivering impressive specific power [1,2]. Supercapacitors are classified based on the mechanism responsible for the charge storage process: (i) an electric double-layer capacitor (EDLC), where charges are physically stored at the EDL within the pores of electrode material and (ii) faradaic charge storage, which can generate pseudocapacitive or battery electrodes [3,4]. In the case of an EDL electrode, surface area plays a crucial role that provides sites for charge storage; thus, the higher the surface area, the higher capacitance can be achieved. However, in pseudocapacitive or battery electrodes, faradaic reactions involve a surface or bulk of electrode material, and charge storage is determined by the capacity of the material with a number of electrons transferred during oxidation and a reduction in ionic species [5].

Nanoporous carbon electrodes find particular suitability for EDLCs due to their highly accessible surface area that facilitates electrostatic or physical charge storage [6,7,8]. This feature facilitates abundant surface sites for electrolyte ions, determining the storage capacity of electrostatic charges. According to the electrode configuration, the supercapacitor devices can be assembled as symmetric, where two carbon electrodes face each other separated by a porous separator, or asymmetric, where one electrode is carbon while the other can be either a pseudocapacitive or battery electrode [9]. Indeed, the asymmetry of electrodes means different charge storage mechanisms at two terminals/polarities of the device differing in capacitance values within a single cell configuration [10,11]. The hybrid electrochemical capacitor is a special variant within the family of supercapacitors that provides high power and energy output simultaneously [12,13]. The hybridization of a battery-like electrode made with iodine and a capacitive electrode is achieved within the capacitor cell while using highly conductive and environmentally friendly aqueous electrolytes [14,15]. The electrodes used in such a hybrid supercapacitor consist of nanoporous carbon with a developed surface and high porosity, where an iodine/iodide redox couple has been shown as a highly reversible and efficient system to achieve high capacity. A well-known method to impregnate iodine in a nanoporous carbon cathode is through the electrochemical oxidation of iodides from an aqueous electrolyte, e.g., aqueous NaI, to deposit solid iodine. However, due to the abundance of iodide ions in the electrolyte, the risks of performance loss related with polyiodides (I_3_^−^, I_5_^−^) formation and shuttling remain high.

Different treatments to design battery electrodes via redox-active species incorporation into the electrolytic solution or confinement into the electrode to exhibit faradaic reactions have been proposed and investigated [16,17]. Halide ion redox couples as charge carrier sources/mediators/additives easily dissolve in aqueous medium and exhibit a fast ion diffusion because of the rapid electrochemical reaction and outstanding reversibility [18]. Furthermore, halide ions such as Br^−^ and I^−^ were confined inside the carbon matrices where strong affinity and adsorption were showcased, thus leading to a good cycle stability of hybrid supercapacitors [19,20]. In this initiative, integrating electroactive iodine to a nanoporous carbon electrode to couple with an EDL electrode offers an economical and reliable source of energy [18,21].

Iodine compounds are mostly soluble in water and can be readily dispersed into the pores of porous carbon to produce a superior rate of performance. The enhancement of capacitance and electrical conductivity induced using iodine is ascribed to iodine chemical interactions with the carbon backbone, leading to charge transfer where iodine easily accepts free electrons available in the carbon network [22]. These charge transfer interactions are so strong that the carbon/iodine complex has been reported to act like a highly conductive wire [23]. Compared to the fabricated hybrid cell using a bromine-treated electrode, the discharge capacitance of the positive electrode modified with iodine retained a higher percentage than the initial value (90.4% vs. 94.9%), in addition to an excellent 99.9% coulombic efficiency, suggesting the successful suppression of polyiodide shuttling. This exceptional performance was attributed to the weakly solvated or the preference of iodide ions and polyiodides to be non-solvated, as indicated by the free energy profiles. 

Future energy storage systems will be hybrid devices accommodating both contributions of battery or battery-like and supercapacitor characteristics to achieve overall higher energy densities without sacrificing the large power outputs [24]. To obtain the required charge equilibrium for stability during the long-term cycling performance of an optimal hybrid device, it is necessary to balance the capacity derived from the redox reaction by appropriately adjusting the mass of active material loaded in each electrode [25]. This is particularly important for iodine-based hybrid supercapacitors, where the carbon electrode’s surface area is less crucial because a high capacity can still be realized with a limited surface area, depending on the capacity of the redox species. Consequently, mass balancing emerges as a crucial parameter for attaining capacitance equilibrium in the device [26]. The charge at the positive and the negative electrode should be equal according to Equation (1) [27].

*q*_+_ = *q*_−_ (1)

therefore, the electrode mass ratio can be calculated using the following [28]:

*C*_+_ *m*_+_ Δ*E*_+_ = *C*_−_ *m*_−_ Δ*E*_−_ (2)

where *C* is the specific capacitance, *m* is the mass, and Δ*E* is the potential window of the electrode. As a result, the disparity of the positive and negative electrode capacitances also allows an expansion of the operating potential window in aqueous systems [29,30], noting that the redox reaction needs to happen when operating with aqueous electrolytes [31]. Thus, the mass balancing of the electrodes is a critical parameter to compensate for the specific capacitance value differences [32]. It surely marks a significant step forward to improve the limited voltage of aqueous-based supercapacitors hindered by the reactions related with oxidation and reduction in water. Further extending the cell voltage has been shown via the water-in-salt approach, which limits the oxidation and reduction reactions of water since almost all the water is utilized in the solvation of ionic species and no free water is available for faradaic processes [33]. 

Herein, a battery-like positive electrode from activated carbon treated with iodine (I_2_) was prepared via a physical impregnation, and the equivalent pristine commercial activated carbon was used as a usual EDL-negative electrode to assemble a hybrid supercapacitor. On this basis, the electrochemical behaviors and performances of the fabricated AC/I_2_//AC, also labelled as carbon_iodine_/carbon_EDL_ hybrid supercapacitors, were examined under concentrated aqueous Li- and Na-ion electrolyte conditions. We introduce a viable method of nanoporous carbon electrode pre-impregnation with iodine to produce a battery electrode to be used in a hybrid supercapacitor. Chemical interactions of solid iodine with the carbon surface and within the pores have been investigated with Raman spectroscopy and XPS, while thermogravimetry supports the finding that iodine is chemically interconnected as an electron acceptor (p-type) dopant within the carbon pores. Electrochemical methods have been used to test electrodes and hybrid supercapacitor devices in aqueous NaNO_3_ and aqueous LiTFSI at 5 mol/L concentration for performance parameters such as energy efficiency, cyclability and energy, and power output. The hybrid supercapacitor in aqueous LiTFSI exhibits stable capacitance and energy efficiency during long-term aging tests at 1.5 V. Resultantly, a balanced charge distribution in electrodes was achieved with an equal 1:1 electrode mass ratio (m+: m−). By using a reference electrode, electrochemical experiments of the three-electrode cells were carried out to monitor the behavior of individual electrodes. The interplay of iodine and the electrolyte ions was also studied to gain an understanding from the resultant performances. Electrode-active nanoarchitecturing utilizing iodine as shown in the present work offers an economical and environmentally friendly approach, without the risks of polyiodides generation, furthering the development of sustainable hybrid supercapacitors.

## 2. Materials and Methods

**Figure 1:**
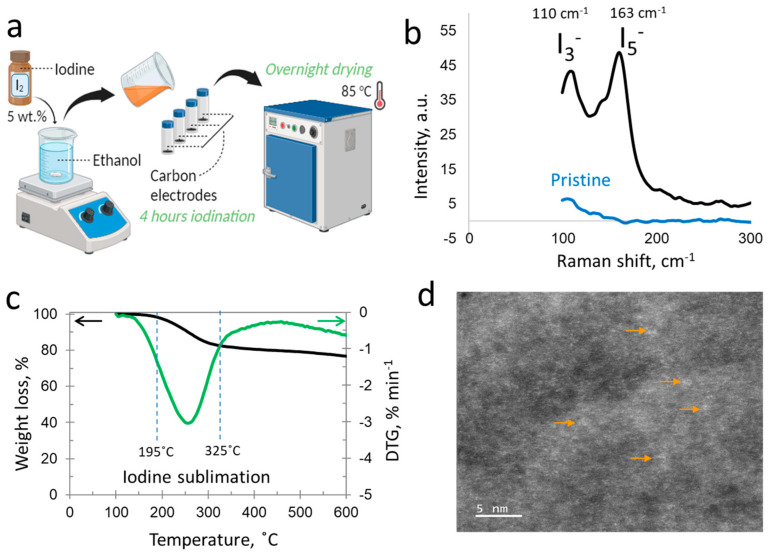
Iodine adsorbed within porous carbon: (**a**) scheme for iodination (physical iodine adsorption) of carbon material, (**b**) Raman spectra of pristine nanoporous carbon and iodinated one exhibiting the presence of I_3_^−^ and I_5_^−^, (**c**) thermogravimetry of carbon electrode impregnated with iodine up to 600 °C, (**d**) TEM of iodinated carbon material, arrows indicating the bright spots dedicated to clusters of iodine adsorbed in the carbon structure.

Lithium bis(trifluoromethanesulfonyl)imide (LiTFSI, 99.99%) was purchased from Ionic Liquids Technologies GmbH (IOLITEC), Heilbronn, Germany and sodium nitrate (NaNO_3_, 98+%) was purchased from Thermo Fisher GmbH, Dreieich, Germany. Electrolytes were prepared by dissolving the salt in deionized water. LiTFSI was dried overnight at 85 °C in a drying furnace before being dissolved to prepare the aqueous electrolytes. I_2_-carbon composites were synthesized via a physical adsorption route, as shown in Figure 1a. In brief, iodine flakes (ACS reagent, ≥99.8%) purchased from Sigma-Aldrich (St. Louis, MO, USA) were added to a beaker of ethanol, constituting 5 wt.% of the ethanol’s total weight, and stirred well until complete dissolution. The resulting iodine–ethanol mixture was then divided among multiple plastic vials, each housing a carbon electrode disk. The carbon electrode immersion in iodine, also referred to as the carbon iodination process, was executed under ambient conditions for 4 h. Upon completion of the iodination, the carbon electrodes were taken out of the vials and sorted into a tray marked with their initial weights for identification. In the final phase, the carbon electrodes underwent overnight drying at 85 °C before they were ready for device assembly.

For the iodine-adsorbed carbon testing and characterization, the structure of the iodinated carbon composite was investigated with a Hitachi Model S-3400 N transmission electron microscope (TEM). Raman analysis was performed using a LabRam 800HR spectrometer (Horiba Jobin Yvon, Osaka, Japan), which features a 1024 × 256 Peltier-cooled CCD and an Olympus BX41 microscope, and this was conducted using a 633 nm He–Ne laser excitation source and 100 s exposure time. Thermogravimetry (TGA) was carried out with the STA 449C Jupitar (Netzsch, Selb, Germany) under helium flow in the 100 °C to 550 °C temperature range at a heating rate of 10 °C per minute. 

A carbon electrode sheet was prepared via mixing 90 wt.% of the YP80-F (Kuraray Chemicals, Okayama, Japan) activated carbon powder (surface area = 2270 m^2^/g, average micropore size = 0.99 nm) with 5 wt.% of carbon black (SUPER C65, Timcal, Bodio, Switzerland) as an electrical conductivity additive, and 5 wt.% of polytetrafluoroethylene/PTFE binder (60 wt.% suspension in water, 3M^TM^, Saint Paul, MN, USA) dispersed in isopropanol. A homogeneous slurry paste was attained after continuous mixing for 12 h at 60 °C and 300 rpm stirring speed; thus, it was ready for manual kneading to roll carbon sheets. Subsequently, the sheets were left overnight in the oven at 85 °C to dry. The diameter of the carbon electrode disk pairs ranged from 6 to 10 mm and weighed between 3 and 10 mg with an average thickness of 110 μm. Adhering to the capacitance unequalization strategy, we achieved electrode mass balancing by constructing a hybrid supercapacitor cell with an equal mass ratio of 1:1 for the positive and negative electrodes. The sample designations, corresponding to the content and active mass ratio following the order of the positive and negative electrodes, are outlined in Table 1.

Supercapacitor devices were assembled using a *T*-type Teflon Swagelok^®^ (Solon, OH, USA) vessel employing an Ag/AgCl reference in 3 mol/L KCl saturation, along with stainless steel as the current collectors and a sheet of microfiber glass filter paper (Whatman, Maidstone, UK, Grade GF/A) as the separator. The electrolyte solutions in the study utilized 5 mol/L aqueous NaNO_3_ and 5 mol/L aqueous LiTFSI. The electrochemical properties of the supercapacitors were assessed using cyclic voltammetry (CV), galvanostatic cycling with potential limitation (GCPL), and potentiostatic charge/discharge. The CV tests were performed in the cell voltage of 0.5 to 1.5 V, with a 2 mV/s sweep rate. The GCPL measurements to determine the potential range of the electrodes were carried out at 200 mA/g, with the current density calculated from the active mass carbon in the two electrodes. Electrochemical measurements were conducted using a VMP3 multichannel potentiostat/galvanostat from Bio-Logic Science Instruments, Seyssinet-Pariset, France, equipped with EC-lab software version 11. Given the imperfect linear behavior observed in galvanostatic charge/discharge curves, the gravimetric capacitance values were derived by integrating the area under the slope of the discharge curves and omitting the ohmic drop. 

## 3. Results and Discussion 

To prepare the battery electrode, firstly, iodine was introduced into the porous network of carbon materials via physical adsorption. Then, one of the two electrodes, namely the positive electrode or cathode, was produced from this iodinated carbon material. The following sections provide details about the physicochemical and electrochemical investigations of the iodinated carbon material, individual electrodes and supercapacitor cells. 

### 3.1. Iodine Physical Adsorption in Nanoporous Carbon

Iodine in its solid form exhibits physical properties just like a metal, such as a crystal structure, hardness and strength. Regarding the electrical properties, iodine requires one electron to complete the outer shell that makes it an electron acceptor. Overall, iodine exhibits semiconductor-like properties with an electrical conductivity of ~1 × 10^−7^ S/cm. The total capacity of iodine is 211 mAh/g. Iodine as a non-polar element is soluble in ethanol or methanol where its solubility can reach up to 20 wt.%. Here, solid iodine is dissolved in pure ethanol at 5 wt.%, followed by the soaking of carbon electrodes in this solution. Carbon electrodes were soaked in iodine solution for 4 h, after which they were shifted to a dryer and dried at 85 °C in order to remove additional ethanol solvent. The amount of adsorbed iodine in the carbon pores was estimated by the careful weighing of electrodes before and after soaking and drying in the solution. The additional mass was counted as iodine adsorbed in carbon, reaching up to 20–35 wt.%, the presence of which was further confirmed using Raman spectroscopy and other methods. Figure 1b shows the Raman spectra of pristine and iodine-adsorbed carbon in the low wavenumber region. The presence of peaks for I_3_^−^ and I_5_^−^ at 110 cm^−1^ and 163 cm^−1^, respectively, confirms the iodine adsorption and its chemical interaction with the carbon electrode. Further, the presence of polyiodide species in the dried carbon electrode also confirms the sustainability of the composite battery electrode material. The evolution and assignment of Raman peaks for the polyiodides has been discussed previously in the case of the iodide-containing aqueous electrolytes, in which either flat electrodes [34,35] or nanoporous carbon [36,37] electrodes were polarized in a certain potential window. Polyiodides formation is indeed reported for the iodine-adsorbed carbons, which concerns the charge transfer reactions of iodine with the carbon material which converts I_2_ to I_3_^−^ and I_5_^−^ due to the electron shift from carbon to iodine. The high peak for pentaiodide compared to triiodide may be dedicated to a higher fraction of iodine already converted to the large structure polyiodide and trapped within the nanoporous network of the carbon electrode. Nevertheless, strong chemical interactions between iodine species and carbon are confirmed using Raman spectroscopy. 

Thermogravimetry conducted up to 600 °C shows a total mass loss of 24 wt.% with the highest rate of loss around 260 °C. However, the major mass loss related with iodine sublimation starts at around 195 °C and takes place up to 300 °C. A higher sublimation temperature than usual for iodine in the iodinated carbon electrode material suggests the entrapment and confinement of iodine within the deep porosity of the material. Further, the sublimation starts from the surface to deep in the pores, causing a continuous mass loss over a wide temperature window. The presence of iodine is further confirmed using the TEM of the iodine-adsorbed electrode, where bright spots indicate contrasting behavior that is related with the molecular iodine. 

### 3.2. Three-Electrode Cell Testing of Symmetric and Hybrid Devices

**Figure 2:**
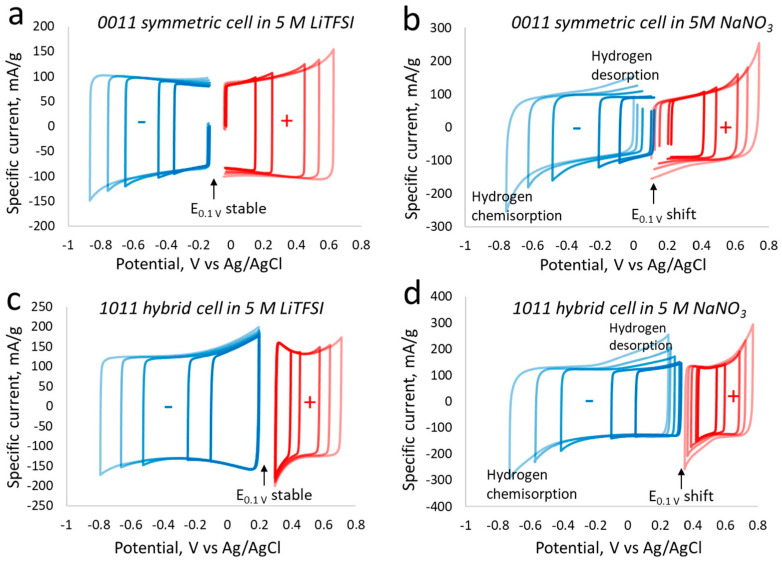
Symmetric versus hybrid cells: cyclic voltammetry conducted on three-electrode cell setup with Ag/AgCl in 3M KCl in voltage range up to 1.5 V, where potential of individual electrode is explored over current versus potential parameters for symmetric (**a**,**b**) and hybrid (**c**,**d**) supercapacitor setup in LiTFSI (**a**,**c**) and NaNO_3_ (**b**,**d**) at concentration of 5 mol/L.

The carbon electrode performance was evaluated in symmetric and hybrid supercapacitor configurations with NaNO_3_ and a LiTFSI-based electrolyte at a concentration of 5 mol/L. For this purpose, symmetric cells equipped with a reference electrode were assembled and tested in these two electrolytes, up to a voltage 1.5 V. CVs in Figure 2a,b display the potential distribution of each electrode of carbon/carbon symmetric cells during charge/discharge. At a narrow voltage of 0.7 V using LiTFSI (Figure 2a), the positive electrode potential reached +0.251 V with a potential window of 0.29 V (Δ*E*+ = 0.29 V) when the cell was charged up to 0.7 V and discharged at 0.1 V. On the other hand, the negative electrode shows Δ*E*− = 0.31 V, with a potential reach to −0.445 V. In the case of NaNO_3_, within a similar voltage window of 0.1 V to 0.7 V, the Δ*E*+ = 0.26 V and Δ*E*− = 0.32 V. In the narrow voltage window, it is clear that both positive and negative electrodes in two electrolyte systems display nearly similar charge/discharge behavior with minor differences in potentials that may be dedicated to a negligible difference in mass balancing and to the different size of ions present in the electrolyte. At the concentration of 5 mol/L, LiTFSI ions are more associated with each other due to the high charge density of lithium compared to NaNO_3_ (ionically more dissociated), which leads to a different behavior during the charging of EDL in the nanopores of the carbon electrode. The difference in individual electrodes at a high cell voltage of 1.5 V is more obvious than at the lower voltage. For example, when the symmetric cells are polarized between 0.1 V and 1.5 V, the potential window of the positive electrode in LiTFSI Δ*E*+ = 0.67 V and in the negative electrode Δ*E*− = 0.718 V, while in NaNO_3_ Δ*E*+ = 0.645 V and in the negative electrode Δ*E*− = 0.774 V. The obvious difference between the two systems working at 1.5 V is the high faradaic redox reactions involved in the NaNO_3_-based electrolyte. The oxidation of electrolytes at the positive electrode and the reduction in water at the negative electrode contribute to the performance loss of symmetric cells in NaNO_3_. On the other hand, thanks to the square-shaped CV exhibiting a smooth charge/discharge LiTFSI-based symmetric cell, the overall performance is expected to be better than the former case. From the symmetric cells, LiTFSI-based setup allows the major charge storage contribution to come from the electric double-layer (EDL) developed at the porous carbon electrodes. Another important finding from this section is the clear difference between the two electrolytes, where LiTFSI shows superior performance due to the strong ionic interactions with water molecules and makes less free water available for contributions in the faradaic reactions. 

Figure 2c,d show the hybrid cells (carbon_iodine_/carbon_EDL_) performance in 5 mol/L LiTFSI and NaNO_3_. The positive electrode exhibits a potential window of 0.148 V, Δ*E*+ = 0.148 V and Δ*E*− = 0.435 V in aqueous LiTFSI when the hybrid cell is charged up to 0.7 V and discharged to 0.1 V. The potential window distribution in aqueous NaNO_3_ electrolyte between positive and negative electrodes is Δ*E*+ = 0.161 V and Δ*E*− = 0.426 V. Two aspects can be derived from the 0.7 V voltage operation of the hybrid cell: (i) the positive electrode in both electrolytes operates in a much narrow potential window than the negative electrode, (ii) the positive and negative electrodes display nearly similar potentials in two electrolytes, positive working in a narrow range of potential, with the negative electrode in a larger potential window. The significantly lower potential window of the positive electrode than the negative one in both electrolytes confirms the redox activity of the iodine/iodine couple, which is adsorbed in the nanopores of the carbon electrode. Additionally, one can see that there are no other faradaic contributions apart from iodine in the voltage up to 0.7 V. At an enlarged voltage of 1.5 V, the hybrid cell in LiTFSI shows Δ*E*+ = 0.4 V and Δ*E*− = 0.98 V and the hybrid cell in NaNO_3_ displays potential values of Δ*E*+ = 0.395 V and Δ*E*− = 0.99 V. The potential windows of individual electrodes in both electrolytes are nearly similar in both electrolytes. However, a noticeable difference is additional faradaic reactions in the case of NaNO_3_ on both positive and negative electrodes. The reactions in aq. NaNO_3_ related with the oxidation of water, which creates surface functional groups on the carbon electrode, are quite significant, as is the reversible hydrogen adsorption and desorption reactions at the negative electrode due to the electrochemical reduction in water. Therefore, concerning the E_0.1V_, the potential of electrodes at a minimum discharge voltage displays an observable shift of E_0.1V_ in aqueous NaNO_3_, but a stable evolution in aqueous LiTFSI.

**Figure 3:**
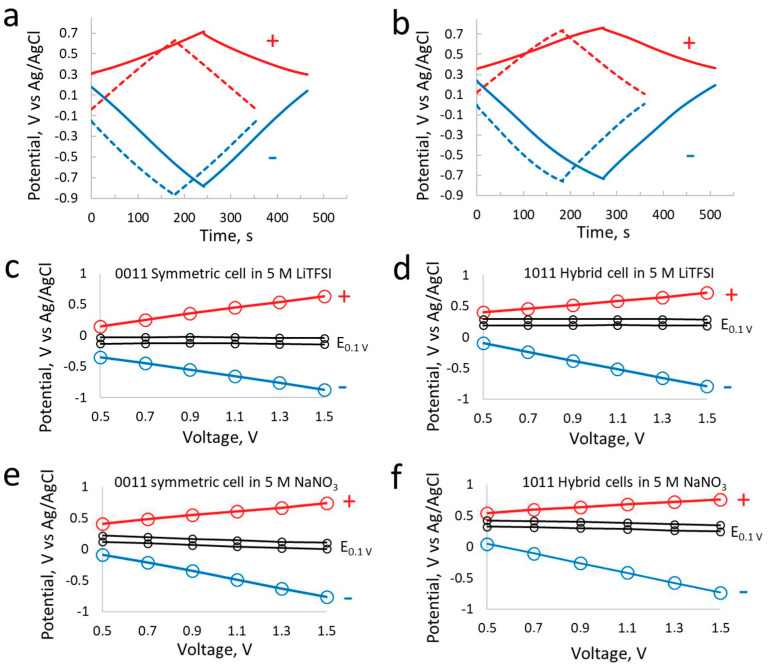
Electrochemistry of individual electrode: exploring the potential window of positive and negative electrodes in symmetric (dashed lines) and hybrid (solid lines) supercapacitor cells (within voltage of 1.5 V) using LiTFSI (**a**) and NaNO_3_ (**b**) at concentration of 5 mol/L and specific current 200 mA/g (per gram of total active carbon material in two electrodes of the cell). Potential excursions of positive and negative electrodes in symmetric (**c**,**e**) and hybrid (**d**,**f**) supercapacitor cells. The potential values are obtained for each selected voltage from galvanostatic charge/discharge at 200 mA/g.

Galvanostatic charge/discharge curves for the symmetric and hybrid supercapacitor cells in aqueous LiTFSI (Figure 3a) and NaNO_3_ (Figure 3b) show linear behavior, especially in the case of carbon/carbon setup, while the symmetric and hybrid cells differ in their E_0.1V_ (minimum potential of electrodes at 0.1 V cell voltage) and the overall upshift of the potential is observed for the hybrid cells. The potential windows of the electrodes in both systems are nearly similar, as discussed for the CVs in the previous paragraph. However, the upshift of the E_0.1V_ is dedicated to the iodine-related redox reactions that drive the positive electrode potential more towards the redox potential of 0.54 V vs. the standard hydrogen electrode (SHE). On the other hand, the minimum potential of the hybrid cell in LiTFSI is more positive than the symmetric one, preventing any risks of hydrogen evolution reactions related with an electrochemical reduction in water. Keeping in view the charge/discharge curves of the negative electrodes in Figure 3a,b, it can be seen that the negative carbon electrodes display non-linear behavior compared to the negative electrodes in LiTFSI. Such an outlook of charge/discharge curves gives the first indication about the energy efficiency and coulombic efficiency, which seem to be higher in LiTFSI than in the NaNO_3_ system. 

Another noticeable difference between the positive electrodes is the narrow potential window in the hybrid cells in both electrolytes, while the potentials are almost similar for the positive and negative electrodes in symmetric cells. The asymmetry of electrodes in hybrid cells appears due to the unequal capacity according to Equations (1) and (2) and thanks to the redox reactions of iodine, which display a high capacity at the positive electrode and consequently a low potential window. The capacity of positive electrodes in hybrid and symmetric cells has been calculated from galvanostatic discharge curves. The positive EDL electrode in aq. NaNO_3_ exhibits 8.5 mAh/g while the battery-like electrode in aq. NaNO_3_ exhibits 12.6 mAh/g. By contrast, the positive electrode in the symmetric cell using aq. LiTFSI shows a capacity of 10.8 mAh/g compared to 12.7 mAh/g for the battery electrode in the same electrolyte. 

Figure 3c–f shows the potential of electrodes versus the cell voltage set for the cut-off voltage of 0.1 V and maximum cell voltage of 1.5 V. These values are extracted from the galvanostatic charge/discharge curves realized at 200 mA/g on cells equipped with the reference electrode. The potential of each electrode was monitored at a selected voltage of either 0.5 V, 0.7 V, 0.9 V, 1.1 V, 1.3 V and 1.5 V. The minimum potential at 0.1 V is displayed for both positive and negative electrodes from 0.5 V to 1.5 V. These figures confirm the continuous narrow potential window of the positive electrodes and the larger potential windows for negative electrodes in hybrid cells at all selected voltages (Figure 3d,f). On the other hand, the shift of E_0.1V_ is clearly observed for the hybrid and symmetric cells in NaNO_3_ (Figure 3e,f), which is absent in the case of aqueous LiTFSI (Figure 3c,d) due to the stability provided by the electrolyte. These shifts of the potential of electrodes are more dominant in the case of aqueous NaNO_3_ due to the highly dissociated ions and the large fraction of available free water that can participate in faradaic reactions, thus strongly influencing the overall supercapacitor performance.

### 3.3. Two-Electrode Cell Testing of Symmetric and Hybrid Devices 

**Figure 4:**
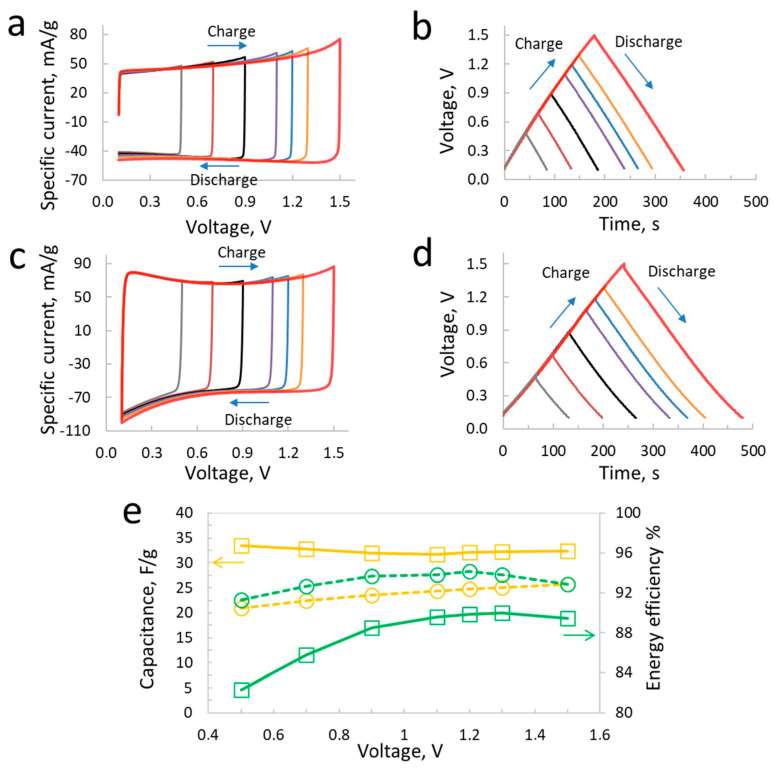
Supercapacitor performance in LiTFSI: cyclic voltammetry profiles (2 mV/s) and galvanostatic charge/discharge (200 mA/g) of symmetric (**a**,**b**) and hybrid (**c**,**d**) supercapacitors in two-electrode setup, (**e**) capacitance (orange lines) and energy efficiency (green lines) of symmetric (dashed lines) and hybrid (solid lines) supercapacitors at various voltages from 0.7 V to 1.5 V.

The two-electrode cell performance of symmetric and hybrid supercapacitors in LiTFSI is displayed in Figure 4. The two-electrode cell cyclic voltammograms (CVs) have been obtained at 2 mV/s from 0.1 V to 1.5 V at different maximum voltages. The CVs of symmetric supercapacitor at this scan rate show square-shaped behavior, which is an indication of the excellent charging of the carbon electrodes with the LiTFSI-based electrolyte. However, some increase in the specific current at the higher voltage of 1.5 V is observed, which is an indication of the onset of the faradaic reactions related with the oxidation of electrolytes, as discussed in the previous section with the individual positive electrode in LiTFSI. The galvanostatic charge/discharge curves in Figure 4b show symmetric behavior at all voltages up to 1.5 V. The symmetry of charge/discharge is well maintained at all cell voltages, which suggests that the faradaic reactions remarked in Figure 4a do not strongly influence the performance at this specific current of 200 mA/g. Thus, at mild charge/discharge rates, the supercapacitor behavior is highly stable after repeated cycles of both CVs and galvanostatic charging. Another noticeable aspect in these curves is the relatively stable behavior of charging/discharging up to 1.3 V; a major change in performance is observed for the last voltage step of 1.5 V, which is due to the electrode potentials exceeding the limits of electrolyte oxidation and reduction at this high cell voltage.

In the case of the hybrid supercapacitor developed using the coupling of an iodinated carbon-based positive electrode and a pristine carbon as a negative electrode, the CVs shown in Figure 4c are square shape, which are more similar to the ones seen for the symmetric supercapacitor. Such a similarity of CVs between the carbon/carbon symmetric supercapacitors and those of the hybrid supercapacitors in iodide-based electrolytes has been previously shown in several works published between 2016 and 2023 [10,11,12,38]. The similar CVs’ outlook is credited to the nearly constant potential window of the positive battery electrode developed using the iodine deposition in the porosity of the carbon electrode. Much like the case of the iodide-based electrolyte, here, we see a higher specific current for the hybrid supercapacitor compared to the symmetric one. However, the shape of the CVs remains square-like, while the galvanostatic charge/discharge curves are symmetric. Thus, the hybrid supercapacitor developed with the carbon_iodine_/carbon_EDL_ configuration displays electrochemical characteristics much like a carbon/carbon supercapacitor, with high capacitance for the former, however. A higher capacitance for the hybrid supercapacitor (33 F/g at 0.5 V and 32 F/g at 1.5 V) compared to the symmetric one (22 F/g at 0.5 V and 24 F/g at 1.5 V) clearly indicates the significant role played by the hybridization of the battery and capacitive electrodes in the hybrid. In the case of the symmetric cell, where two carbon electrodes exhibit nearly similar capacitance, the net cell capacitance is half of the capacitance of a single electrode. On the other hand, as the capacitance of the positive battery electrode is much higher than the negative electrode, the negative capacitive electrode determines the net capacitance of the hybrid cell and it is higher than the case of the symmetric supercapacitors. 

Figure 4e shows a comparison of energy efficiency values evaluated at different voltages of symmetric and hybrid supercapacitor cells. Energy efficiency is a reliable parameter that indicates the state-of-health of the supercapacitor across various voltages scanned via galvanostatic charge/discharge curves (in this case at 200 mA/g). In order to evaluate the energy efficiency, the area under the discharge curves is divided to the area under charge curve at a given voltage. One can see that the symmetric supercapacitor at 5 mol/L LiTFSI exhibits a high energy efficiency of ~92% compared to the hybrid supercapacitor, with 82% at 0.5 V and 89% at 1.5 V. Thus, the relatively low energy efficiency of the hybrid carbon_iodine_/carbon_EDL_ supercapacitor compared to the symmetric one is due to the continuous involvement of redox reactions at the positive battery electrode related to the presence of the iodine species. An increasing energy efficiency from 0.5 V to 1.3 V for the hybrid supercapacitor can be explained with reference to individual electrode performance, where the positive electrode potential window increases and does not exhibit additional faradaic reactions at a high voltage, as shown in Figure 2, whereas the negative electrode potential window shows an almost similar charge/discharge pattern independent of the scanned voltage window, which is partly due to the stronger electrolyte ions association; consequently, there is less free water available for the faradaic reaction. Comparing the specific energy values, the hybrid supercapacitor in aq. LiTFSI exhibits 9.1 Wh/kg compared to 7.2 Wh/kg for the symmetric supercapacitor at a relatively low power of 0.3 kW/kg. 

**Figure 5:**
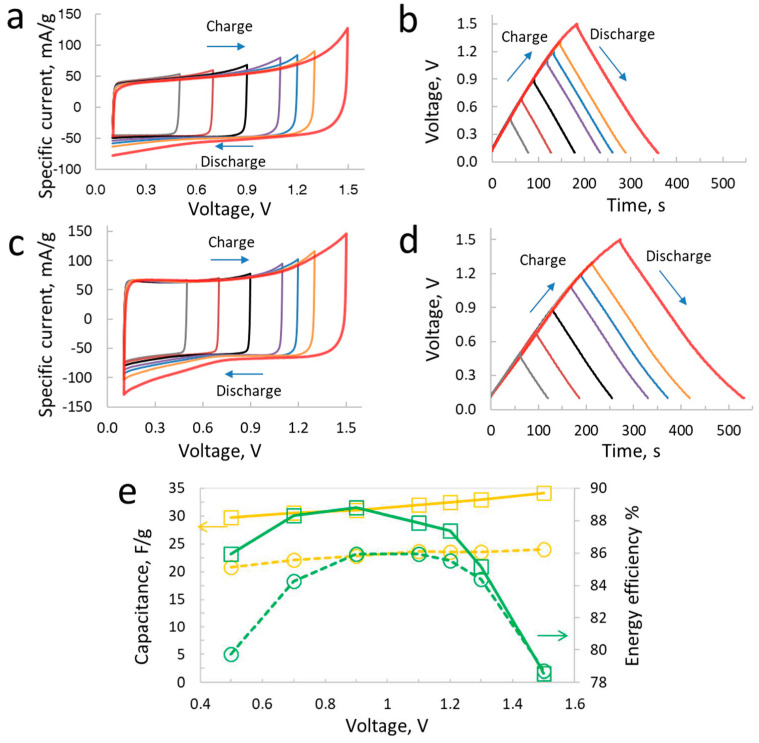
Supercapacitor performance in NaNO_3_: cyclic voltammetry profiles (2 mV/s) and galvanostatic charge/discharge (200 mA/g) of symmetric (**a**,**b**) and hybrid (**c**,**d**) supercapacitors in two-electrode setup, (**e**) capacitance (orange lines) and energy efficiency (green lines) of symmetric (dashed lines) and hybrid (solid lines) supercapacitors at various voltages from 0.7 V to 1.5 V.

The two-electrode cell performance of symmetric and hybrid supercapacitors in 5 mol/L NaNO_3_ is shown in Figure 5. CVs and galvanostatic charge/discharge curves in this case display asymmetric behavior in the carbon/carbon configuration. When compared to the previous case of LiTFSI, the CVs of the symmetric supercapacitor in NaNO_3_ show an increasing specific current as the voltage increases up to 1.5 V. On the other, in the case of galvanostatic charge/discharge curves, the shape is linear at lower voltages, but an observable bend in the charging curve at 1.5 V suggests that the oxidation and reduction reactions play a significant role in the positive and negative electrodes. Although the galvanostatic charge/discharge curves at 1.3 V in Figure 5 are symmetric, the real onset of faradaic reactions and consequently a shift in the charging characteristics are clearly observed at 1.5 V. From the individual electrodes’ performance data, it can be recalled that faradaic reactions related to the hydrogen adsorption caused through the electro-reduction of water are highly reversible. Therefore, hydrogen adsorption/desorption reactions at the negative electrode do not seriously affect the state-of-health of the symmetric supercapacitor. By contrast, the positive electrode in this symmetric supercapacitor operating 5 mol/L NaNO_3_ shows an increasing specific current at the forward scan, while at the reverse scan this current is not extracted to a similar extent, which suggests that some irreversible oxidation reactions take place at the positive carbon electrode. The oxidation of the positive carbon electrode in aqueous electrolyte-based supercapacitors has been reported to cause a blockage of porosity, leading to performance decay at high voltages [39,40]. Thus, the positive electrode oxidation in aqueous NaNO_3_-based aqueous electrolyte may cause performance loss, affecting the efficiency and cyclability.

By comparison, the carbon_iodine_/carbon_EDL_ hybrid supercapacitor in aqueous NaNO_3_ shows square-shaped CVs at 2 mV/s and symmetric charge/discharge curves with an applied current of 200 mA/g at a low voltage window. This indicates that within the limited voltage window of 1.0–1.2 V, the hybrid supercapacitor displays similar electrochemical characteristics as the symmetric cell. However, when the voltage is further increased to 1.3 V and up to 1.5 V, the contributions related with faradaic reactions at the negative electrode due to the hydrogen adsorption strongly influence the performance. One can see that, from the CV and charge/discharge curve in Figure 5c,d, the specific current increase when it reaches 1.5 V shows reversible behavior at the reverse scan. At this voltage, the galvanostatic charge/discharge curve also shows a bend, reaching the maximum value during charging with a reversible pattern at a low voltage during discharge. 

The capacitance of the symmetric carbon/carbon supercapacitor in aqueous NaNO_3_ is 21 F/g at 0.5 V and increases to 24 F/g at 1.5 V. For the hybrid supercapacitor, the capacitance of 30 F/g is achieved at 0.5 V compared to 34 F/g at 1.5 V. Thus, a higher capacitance for the hybrid supercapacitor is observed compared to the symmetric one at all voltage values. The energy efficiency of the symmetric supercapacitor is only 80% at 0.5 V, which is less than the hybrid supercapacitor showing 86% at the same voltage. The efficiency values for the hybrid supercapacitor increase up to 1.0 V, reaching ~89% before decreasing again to 78.5% at 1.5 V. A decrease in energy efficiency in the hybrid supercapacitor is due to the faradaic contribution at the negative electrode that is not fully reversible. This is the same pattern observed in the symmetric supercapacitor, when the energy efficiency is also 78.5% at 1.5 V. This indicates a similar behavior in the negative electrode in symmetric and hybrid supercapacitors, which is dictated by the hydrogen adsorption and desorption reaction, which in turn plays a crucial role in determining the performance of supercapacitors. Indeed, the ionic properties of the NaNO_3_ electrolyte also affect the performance of supercapacitors, indicated by a much lower energy efficiency compared to aqueous LiTFSI at 1.5 V. Interestingly, a major downward shift in the energy efficiency occurs at 1.5 V, as these values are comparable at 1.3 V to the supercapacitors in aqueous LiTFSI. When comparing the energy performance, the hybrid supercapacitor in aq. NaNO_3_ exhibited 8.9 Wh/kg compared to the symmetric supercapacitor showing 6.8 Wh/kg at a specific power of 0.3 kW/kg. 

### 3.4. Performance Testing in Self-Discharge and High-Voltage Floating

**Figure 6:**
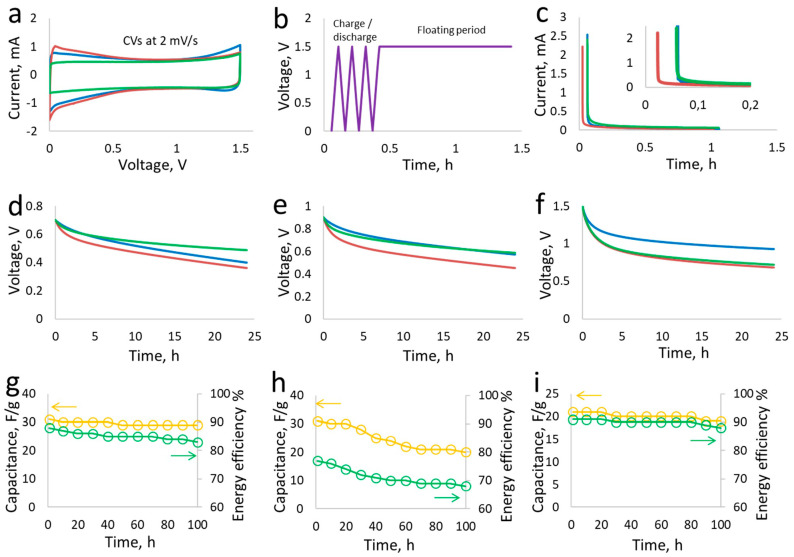
Self-discharge and floating tests: (**a**) CVs at 2 mV/s, green—symmetric cell in 5 mol/L LiTFSI, blue—hybrid cell in LiTFSI and red—hybrid cell in NaNO_3_, (**b**) protocol for the charge/discharge followed by the floating period or voltage hold, (**c**) leakage current profiles of supercapacitors during floating period, (**d**–**f**) self-discharge evaluated for 24 h after 1 h of voltage hold at 0.7 V, 0.9 V and 1.5 V, plots of capacitance and energy efficiency versus total floating period of 100 h at 1.5 V (capacitance and efficiency calculated after each floating period of 10 h) for the (**g**) hybrid supercapacitor in LiTFSI, (**h**) hybrid supercapacitor in NaNO_3_ and (**i**) symmetric supercapacitor in LiTFSI.

Symmetric and hybrid supercapacitors assembled with 1:1 electrode mass ratio in aqueous LiTFSI were selected for long-term potentiostatic floating and self-discharge tests. For comparison, a hybrid supercapacitor assembled in aqueous NaNO_3_ has been used. The CVs in Figure 6a are compared up to 1.5 V for the symmetric and hybrid supercapacitors especially constructed for the purpose of self-discharge and floating tests. 

The symmetric supercapacitor shows a square-shaped CV with a charge stored mainly at the EDL, while the CVs of hybrid supercapacitors suggest the involvement of redox reactions related with iodine, which is indicated by an enhanced current. These supercapacitors were charged for an hour continuously at a constant voltage before shifting to open circuit voltage in order to determine self-discharge. The purpose of voltage hold is to properly charge the bulk electrode porosity and distribute charges within the electrode matrix. Figure 6d–f show the self-discharge comparison at a low voltage and at 1.5 V, where the hybrid supercapacitor maintains good charging characteristics during 24 h of the open circuit voltage period compared to the hybrid supercapacitor in NaNO_3_. The symmetric supercapacitor, on the other hand, initially displays very good self-discharge behavior, maintaining a high voltage for 24 h. During the long-term floating period of 100 h, the capacitance and energy efficiency of the supercapacitors is compared in Figure 6g–i. The hybrid supercapacitor in LiTFSI as well as the symmetric one in LiTFSI show an excellent performance of capacitance and energy efficiency, which mostly remains stable for the entire period of floating. A decay of capacitance in the case of the hybrid supercapacitor using aqueous NaNO_3_ can be ascribed to the presence of additional faradaic reactions at positive and negative electrodes, as discussed in previous sections. From the potentiostatic floating data, the loss of capacitance comparison between the hybrid supercapacitor in aq. NaNO_3_ and the symmetric supercapacitor in aq. LiTFSI indicates capacitance decay due to polyiodides shuttling in the former. From these data, an additional capacitance loss of ~30% in NaNO_3_ (estimated between 20 h and 50 h of floating) can be related to polyiodides, which is absent in both symmetric and hybrid supercapacitors using LiTFSI. 

The tested model of hybrid supercapacitors with aq. NaNO_3_ and aq. LiTFSI displayed 611 Wh and 429 Wh, respectively, as the total amount of energy stored. Taking into account the mass of active electrode material between 0.013 g and 0.019 g, the further mass of components such as a separator, binder (up to 5 wt.%) and cell casing can be scaled, indicating an overall low specific energy. Nevertheless, the main advantages of the reported hybrid supercapacitor are high power delivery, which at low currents of 200 mA/g reaches up to 0.3 kW/kg, and its low cost of components and cell assembly that do not require any high vacuum conditions, making it suitable for large-scale applications. 

## 4. Conclusions

The physical iodination of nanoporous carbon electrodes represents an eco-friendly and low-cost method to produce battery-like electrodes with an ultra-long cycle life in aqueous electrolytes. The chemical interactions of iodine adsorbed in the carbon nanopores are confirmed by the generation of iodide species detected using Raman spectroscopy. The charge transfer between carbon and iodine enables a significantly high performance of this composite material for charge storage applications. The high capacity of iodine-adsorbed carbon electrode is balanced through the proper equalization of the active mass of the carbon electrode in the counter electrode, which is crucial for the assemblage of a durable supercapacitor device. The iodinated carbon electrode used to fabricate the carbon_iodine_/carbon_EDL_ hybrid supercapacitor displays excellent performance in aqueous LiTFSI electrolyte, owing to strong ionic association in this electrolyte and less free water available for oxidation and reduction reactions, as well as a reduced shuttling effect. The hybridization or coupling of a battery-like and capacitive electrode in a single device using highly concentrated electrolytes offers a viable method for enhancing supercapacitor energy density while maintaining a long cycle life. Investigations of the charging behavior of carbon electrodes and a full device prove that iodine adsorbed in the positive battery electrode is well-confined in the porosity of carbon material, and the risks of the shuttling of polyiodides is significantly reduced to allow excellent performance under potentiostatic floating at high voltage. Given the relatively low specific energy output, such hybrid supercapacitors could be useful in grid-level stationary applications. Future attempts in this research will be focused on enhancing the discharge capacity of iodinated carbon electrodes to further improve the energy density of hybrid supercapacitors. 

## Figures and Tables

**Table 1:** Device labelling: positive and negative electrodes with mass ratio of 1:1 were used for assembling symmetric and hybrid supercapacitor cells.

Iodine Treatment ^†^		Mass Ratio ^‡^		Sample/Configuration
Positive (+)	Negative (−)	Positive (+)	Negative (−)	
0	0	1	1	0011/Symmetric
1	0	1	1	1011/Hybrid

^†^: A binary indicator of 0 or 1 denotes the absence or presence of iodine (subjected to iodination or not) in the respective electrode. ^‡^: The indicated number represents the value/proportion (i.e., ‘cathode = 1, anode = 1’ denotes a mass ratio of 1:1 between the cathode and anode).

## Data Availability

The raw data supporting the conclusions of this article will be made available by the authors on request.
